# Identification of Five Serum Protein Markers for Detection of Ovarian Cancer by Antibody Arrays

**DOI:** 10.1371/journal.pone.0076795

**Published:** 2013-10-08

**Authors:** Weidong Jiang, Ruochun Huang, Chaohui Duan, Liwu Fu, Yun Xi, Yuebo Yang, Wei-Min Yang, Dongzi Yang, Dong-Hua Yang, Ruo-Pan Huang

**Affiliations:** 1 RayBiotech, Inc, Norcross, Georgia, United States of America; 2 RayBiotech, Inc, Guangzhou, China; 3 Department of Clinical Laboratory, the Second Affiliated Hospital of Sun Yat-sen University, Guangzhou, China; 4 The Affiliated Cancer Hospital of Sun Yat-sen University, Sun Yat-sen University, Guangzhou, China; 5 Department of Clinical Laboratory, the Third Affiliated Hospital of Sun Yat-sen University, Guangzhou, China; 6 Department of Obstetrics and Gynecology, the Third Affiliated Hospital of Sun Yat-sen University, Guangzhou, China; 7 Department of Gynecology, Wuxi Maternal and Child Health Hospital, Wuxi, China; 8 Department of Obstetrics and Gynecology, the Second Affiliated Hospital of Sun Yat-sen University, Guangzhou, China; 9 Biosample Repository, Fox Chase Cancer Center, Philadelphia, Pennsylvania, United States of America; 10 South China Biochip Research Center, Guangzhou, China; The University of Kansas Medical center, United States of America

## Abstract

**Background:**

Protein and antibody arrays have emerged as a promising technology to study protein expression and protein function in a high-throughput manner. These arrays also represent a new opportunity to profile protein expression levels in cancer patients’ samples and to identify useful biosignatures for clinical diagnosis, disease classification, prediction, drug development and patient care. We applied antibody arrays to discover a panel of proteins which may serve as biomarkers to distinguish between patients with ovarian cancer and normal controls.

**Methodology/Principal Findings:**

Using a case-control study design of 34 ovarian cancer patients and 53 age-matched healthy controls, we profiled the expression levels of 174 proteins using antibody array technology and determined the CA125 level using ELISA. The expression levels of those proteins were analyzed using 3 discriminant methods, including artificial neural network, classification tree and split-point score analysis. A panel of 5 serum protein markers (MSP-alpha, TIMP-4, PDGF-R alpha, and OPG and CA125) was identified, which could effectively detect ovarian cancer with high specificity (95%) and high sensitivity (100%), with AUC =0.98, while CA125 alone had an AUC of 0.87.

**Conclusions/Significance:**

Our pilot study has shown the promising set of 5 serum markers for ovarian cancer detection.

## Introduction

Ovarian cancer represents the third most frequent cancer and is one of the leading causes of cancer death among females in the United States and Europe [1-3]. Most symptoms of ovarian cancer are vague and similar to those often experienced with more common, non-life–threatening health conditions; these might include abdominal swelling or bloating, pelvic pain or discomfort, lower back pain, loss of appetite or feeling full quickly, persistent indigestion, gas or nausea and changes in bowel or bladder habits. As a result, almost 80% of ovarian cancer patients are diagnosed at later stages. Unfortunately, the 5-year survival rate for patients with clinically advanced ovarian cancer is only 15% to 20%, in striking contrast to a 5-year survival rate of over 90% for patients with stage I disease. Therefore, it is urgent to discover and develop biomarkers for ovarian cancer screening and early detection.

Currently, CA-125 and imaging are the 2 most common approaches for ovarian cancer screening tests. However, these 2 markers, either used alone or in combination, are not useful screening or diagnostic purposes due to low specificity and/or sensitivity. For example, serum CA-125 has been shown to have a sensitivity of >98% but a specificity of only 50-60% for early-stage disease [4-6].

Multiple studies have been reported to identify serum ovarian cancer biomarkers using multiplex antibody array technology [7-9]. Dr. Lokshin’s group identified a group of 6 serum protein markers, including interleukin-6 (IL-6), interleukin-8 (IL-8), epidermal growth factor (EGF), vascular endothelial growth factor (VEGF), monocyte chemoattractant protein-1 (MCP-1), and CA-125, which displayed significant difference in serum concentrations between ovarian cancer and control groups with 84% sensitivity at 95% specificity [7]. Dr Gil Mor’s group identified a panel of 6 biomarkers, CA-125, osteopontin (OPN), insulin-like growth factor 2 (IGF-II), macrophage migration inhibitory factor (MIF), leptin and prolactin, which demonstrated a sensitivity of 95.3% and a specificity of 99.4% for the detection of ovarian cancer [8]. Using human biotin-based antibody arrays, we screened the serum expression profiles of 507 proteins in serum samples from 47 patients with ovarian cancers, 33 patients with benign ovarian masses and 39 healthy, age-matched controls and identified significant differences in protein expression between normal controls and patients with ovarian cancer (*P*<0.05). By classification analysis and split-point score analysis of these 2 groups, a 6-marker panel of proteins, which consisted of interleukin-2 receptor alpha (IL2Rα), endothelin, osteoprotegerin (OPG), vascular endothelial growth factor D (VEGF-D) and betacellulin (BTC), can be used to distinguish ovarian cancer patients from normal subjects [9]. These studies strongly suggest that antibody array technology has shown great promise in the discovery and development of serum ovarian cancer biomarker profiles and strongly suggest that serum cytokine panels may be useful as biomarkers for early detection of ovarian cancers.

In this study, we used our 174-marker, sandwich ELISA-based antibody array panels to screen serum samples from 34 ovarian cancer patients and 53 normal healthy subjects in order to identify a serum protein marker panel for detection of ovarian cancer.

## Results

### Validation of 174-marker semi-quantitative cytokine arrays (Figures 1, 2)

**Figure 1:**
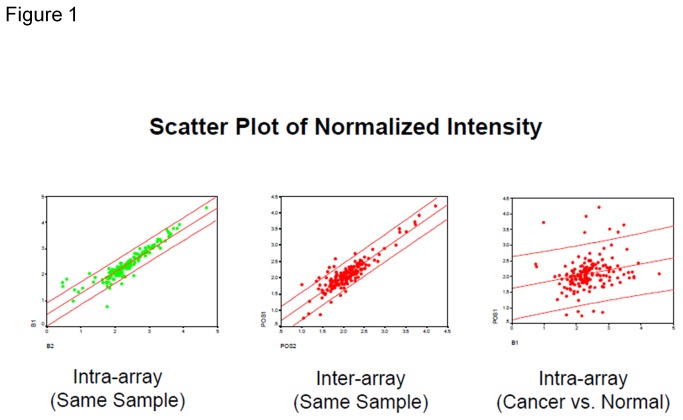
Scatter plot for normalized intensity of 174marker antibody arrays. Panel A (left) shows strong intra-assay correlation (same sample assayed on the same glass slide, tested on the same day); Panel B (middle) shows strong inter-assay correlation (same sample assayed on different glass slides, tested on different days); Panel C (right) shows poor correlation between cancer and normal samples assayed on the same glass slides, tested on the same day.

**Figure 2:**
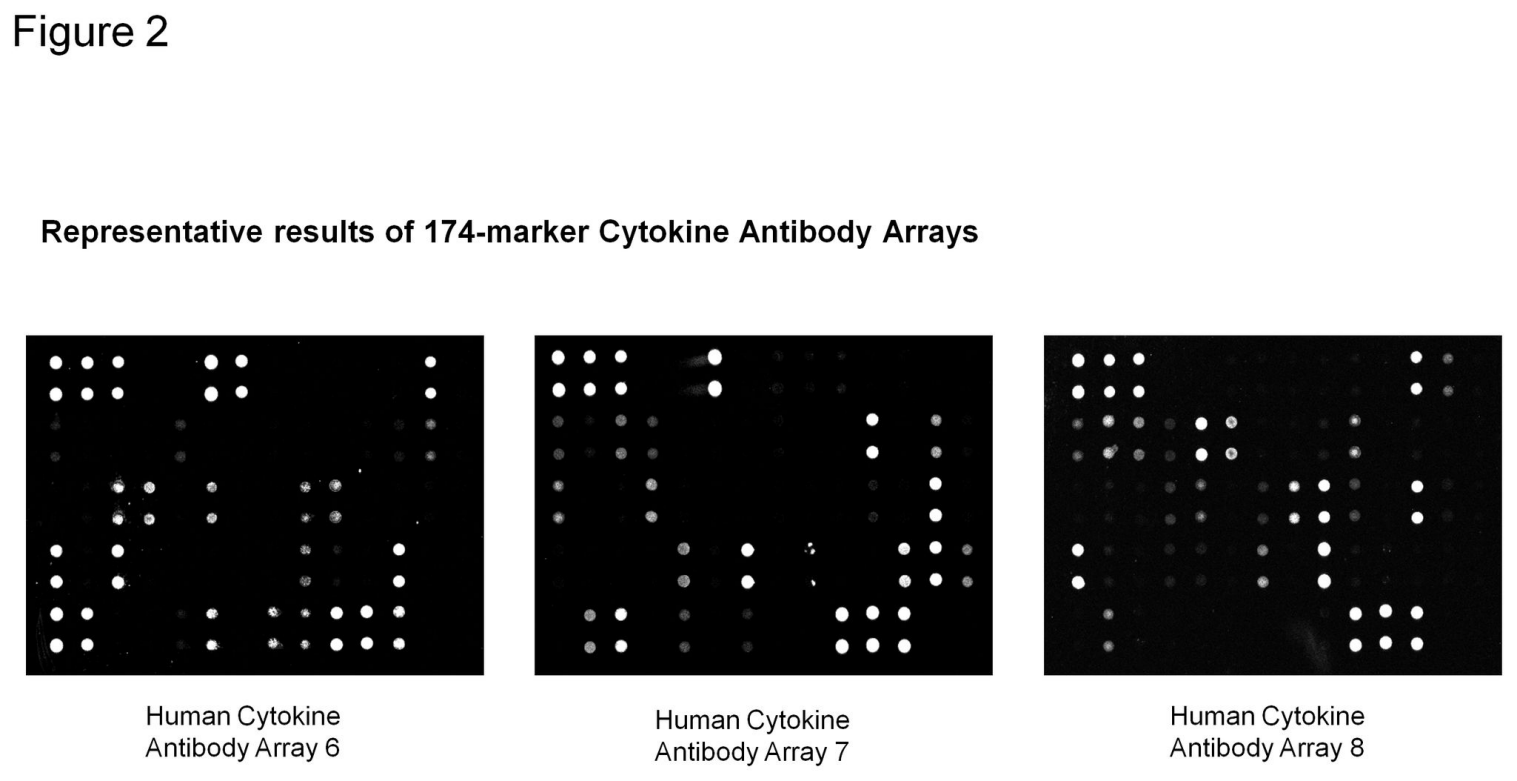
Representative results for 174-marker antibody arrays. Panel A (left) shows representative fluorescent signal images for array G6; Panel B (middle) shows representative fluorescent signal images for array G7; Panel C (right) shows representative fluorescent signal images for array G8.

In this study, we applied antibody array technology to determine the expression profiles of 174 cytokines in the serum from ovarian cancer patients and age-matched healthy normal controls. Cytokines in this study included anti-inflammatory cytokines, proinflammatory cytokines, growth factors, angiogenic factors or chemotactic cytokines, among others. Some of these cytokines reportedly are altered in ovarian cancer patients from our own studies and literature, but our broad screen of 174 proteins also included many other types of markers as part of an “unbiased” approach of using high-content, high-throughput cytokine antibody arrays to profile the cytokine levels from ovarian cancer patients’ serum with the goal of identifying potential diagnostic biomarkers.

First, we further determined the reproducibility of the assay in the analysis of human serum using scatter-plot analysis. Intra-slide reproducibility for the glass-slide–based arrays was assessed by testing replicate aliquots of the same samples with two sub-arrays printed on the same slide and assayed at the same time. The inter-slide reproducibility was determined using two different slides printed with the same arrays were assayed using duplicate aliquots of the same samples on two different days. The Pearson correlation coefficients for intra-slide and inter-slide reproducibility were 0.923 (*P*<0.001) and 0.899 (*P*<0.001) respectively, suggesting high reproducibility of the assay. In contrast, the Pearson correlation coefficient for cancer vs. normal samples were 0.226 (*P*<0.005), suggesting that the cancer samples and normal samples are from two different populations.

Next, serum from a total of 34 ovarian cancer patients and 53 healthy controls were assayed for expression levels of 174 cytokines with the goal of discovering new diagnostic markers for ovarian cancer. These serum samples were mainly obtained from our collaborators and were age- and sex-matched (Table 1). Human Cytokine Antibody Arrays were used to profile expression patterns for 174 cytokines in all 87 patients’ serum samples. The signal intensity is proportional to the expression level of an individual protein in each sample. The array data were then normalized based on the average positive control signal intensity of each array. The median signal intensities of every spot were then corrected for local background. To establish a signal threshold, signal intensity cut-off value was determined by+/-2SD of 10 buffer blank control signal intensities, where the arrays were incubate with blocking buffer instead of patient’s serum samples. Any values exceeding the signal threshold were considered as real signals (i.e., a positive detection of the cytokine). Values lower than the signal cut-off were assigned a value of 1. If measured signal intensity values from all samples for a particular cytokine were 1, those cytokines were removed from the list for further analysis.

**Table 1:** Study population characteristics.

	**Ovarian Cancer**	**Healthy Control**
***Total Number***	**34**	**53**
**Mean Age**	**61.7**	**51.2**
**Median Age**	**66**	**56.2**
**Age Range**	**26-79**	**28-79**
***Cancer Characteristis***		
***Histology***		
**Serous Adenoocarcinoma**	**29**	
**Mucous Adenocarcinoma**	**4**	
**Germline tumor**	**1**	
***Stage***		
**Stage I**	**4**	
**Stage II**	**3**	
**Stage III & IV**	**25**	
**NA**	**2**	

### Identification of serum protein markers by artificial neural network analysis (Figure 3)

**Figure 3:**
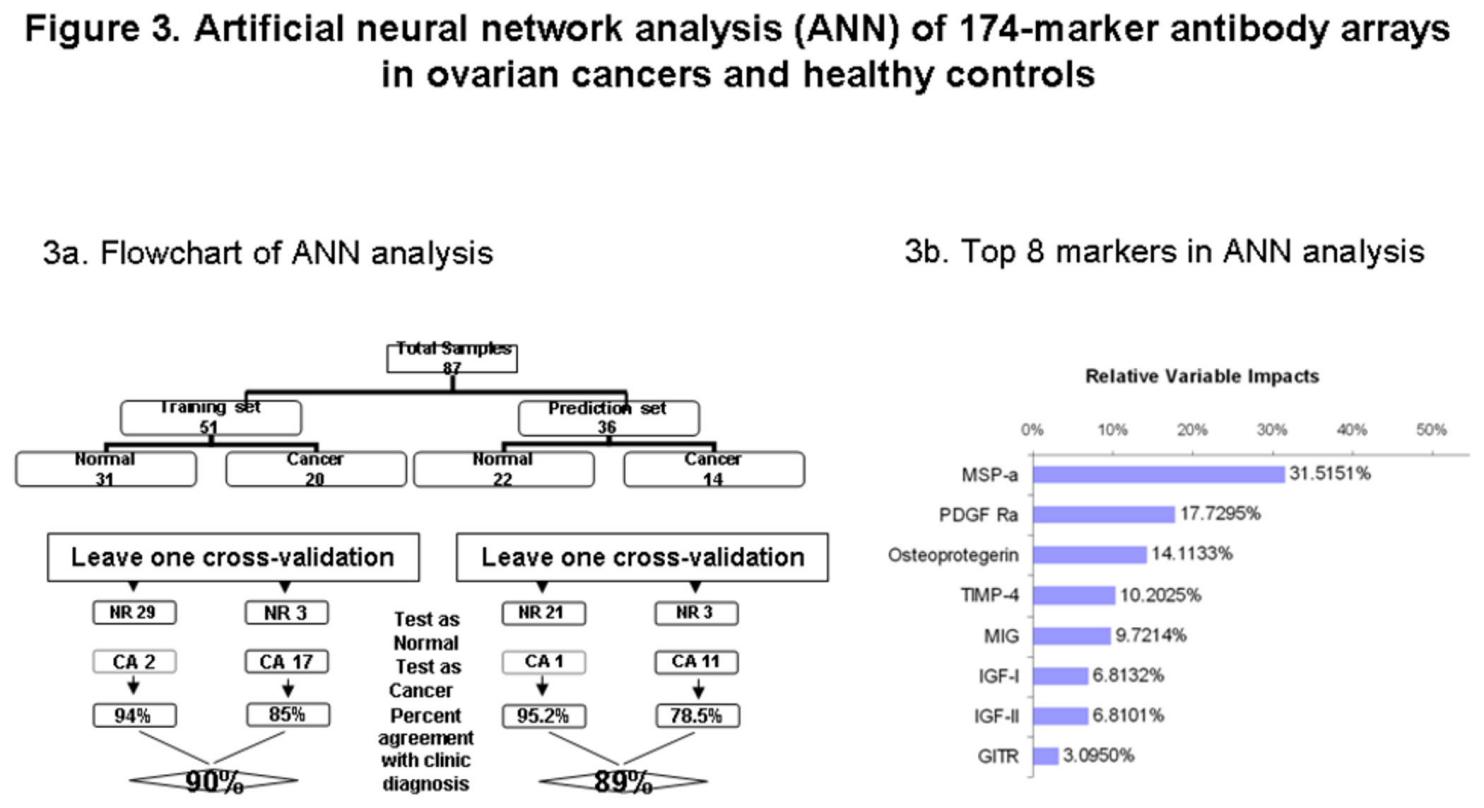
Artificial neural network analysis of 174-marker antibody array results in ovarian cancers and healthy controls. 3a. Artificial neural network analysis of 174-marker antibody array results comparing ovarian cancers and healthy controls. Samples representing both the training set and prediction set are depicted in the graph. 3b. The top 8 markers with the greatest impact in artificial neural network analysis of 174-marker antibody arrays in ovarian cancers and healthy controls are presented.

After normalization and filtration, the data were then subjected to artificial neural network (ANN) analysis. The signal intensity data for individual patients were randomly divided into the training set (N= 51) or prediction set (N=36). In prediction discovery phase, the training set was analyzed using leave-one cross-validation approach. Through this analysis, a total of 8 predictors were identified. These 8 predictors were then used to predict the disease status in prediction set. The correct agreement of predicted disease status using the 8-marker panel with clinical diagnosis in the training set and prediction set was 82% and 80% respectively.

### Identification of 5-marker panel for detection of ovarian cancer (Figures 4 and 5)

**Figure 4:**
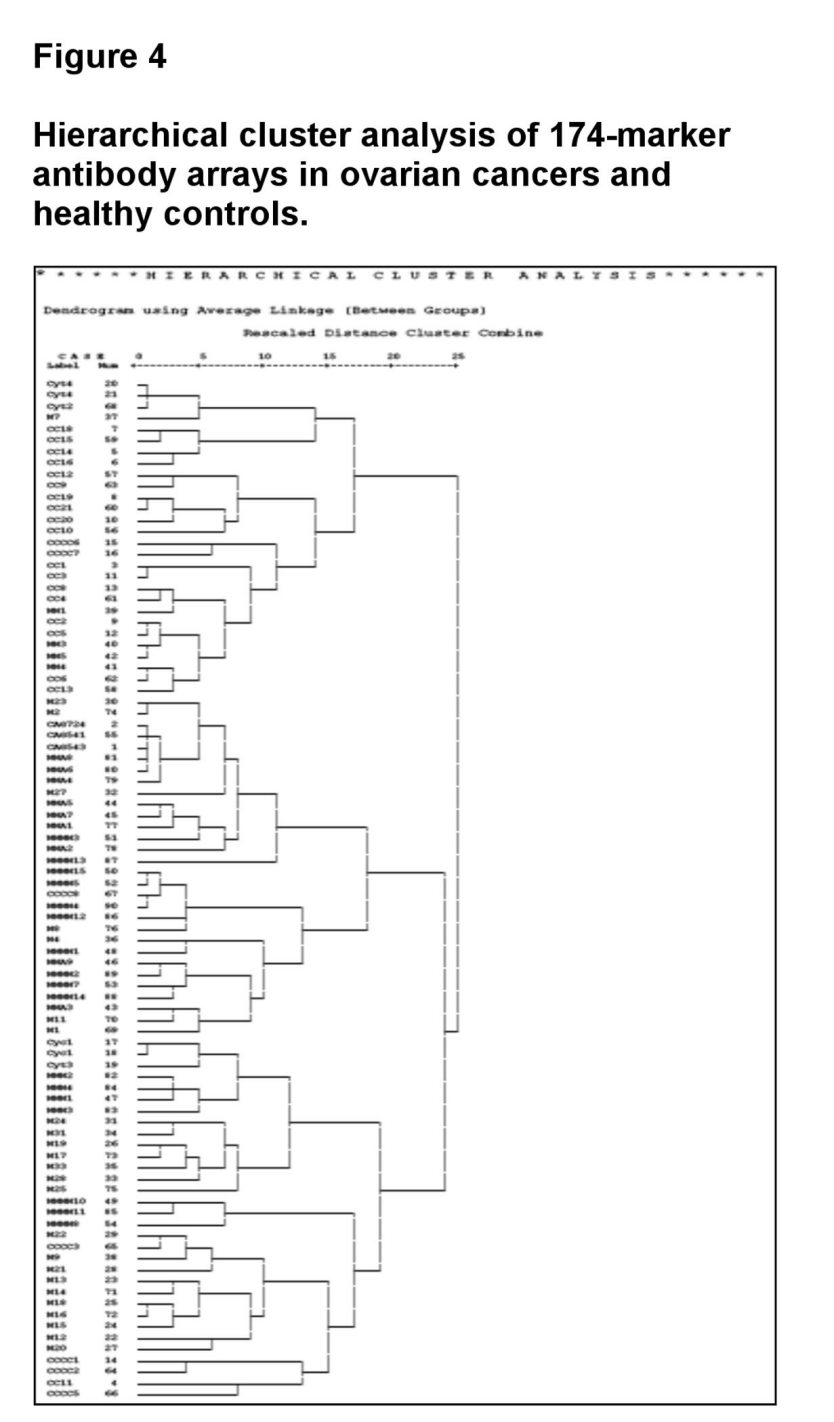
Hierarchical cluster analysis of 174-marker antibody arrays in ovarian cancers and healthy controls.

**Figure 5:**
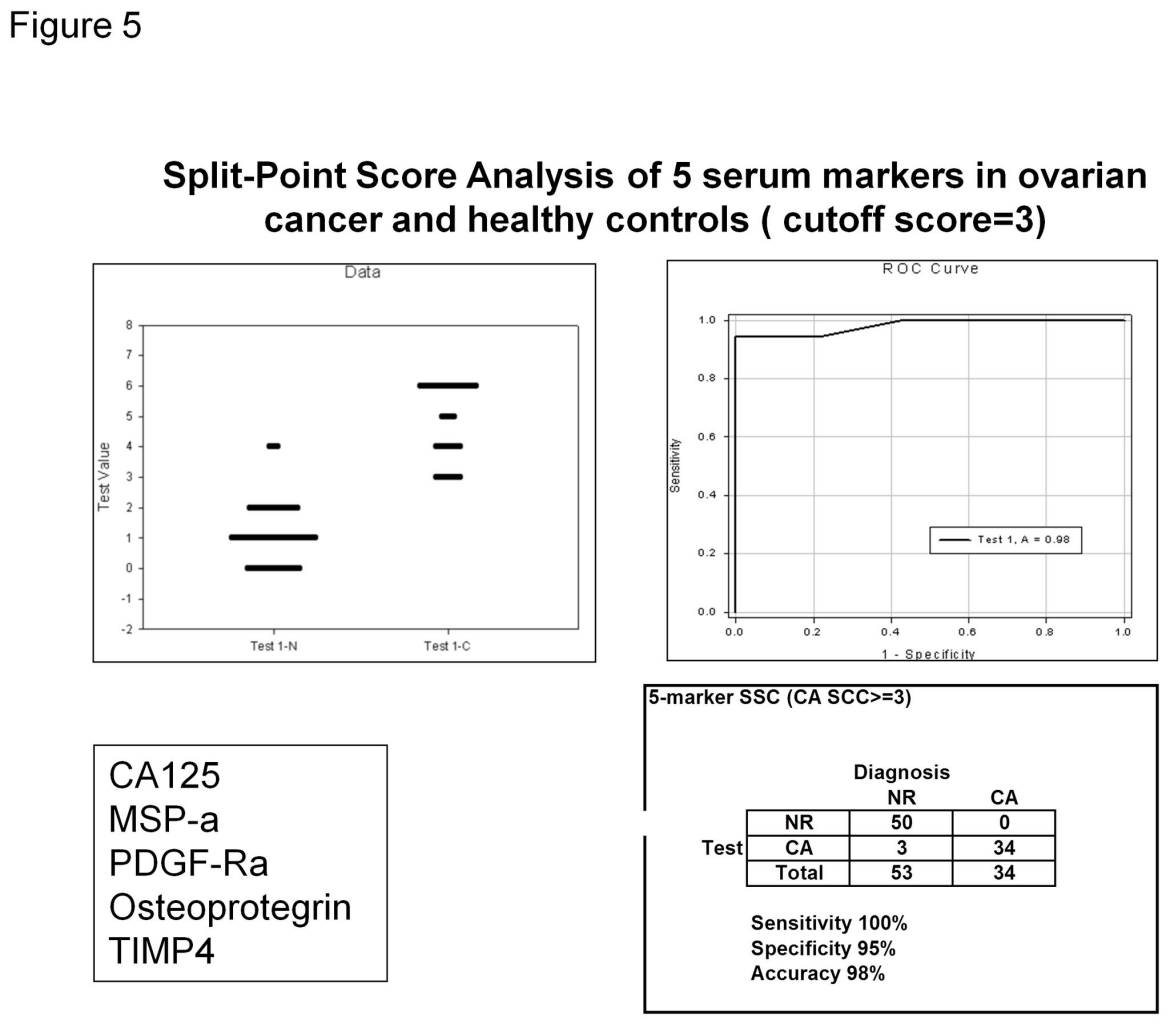
Split-Point Score Analysis of 5 serum markers in ovarian cancer and healthy controls. Panel A (top left): Dot histogram plot with 5-analyte split-point score classification of sera from healthy control (N) and ovarian cancer (CA). Correctly classified normal serum samples should have a score of 0 to 2, whereas samples from ovarian cancer patients should have a score of 3 to 5; Panel B (top right): The ROC curve for 5-marker panel of split-score analysis of ovarian cancer vs. healthy controls. The ROC is the curve plotted of sensitivity (true positive) against 1-specificity (false positive) values; Panel C (bottom right): Table using five-marker split-point score to classify ovarian cancer patients. A cut-off score of 3 was used.

Next, of these 8 markers, we chose 4, macrophage stimulating protein alpha (MSP-alpha), tissue inhibitor of metalloproteinases-4 (TIMP-4), platelet derived growth factor receptor alpha (PDGF-R alpha), and osteoprotegerin (OPG), for hierarchal cluster analysis using SPSS software. Using the 4-marker panel above, 83% of samples were correctly identified (95% of healthy controls and 62% of ovarian cancers).

Finally, all 87 samples were analyzed by the above identified 4 serum markers plus CA125 using split-point score analysis. Using the cutoff score of 3, 100% ovarian cancer and 95% healthy control samples were correctly identified, giving the total correct agreement of 96.6%.

Since CA125 is the most widely used marker for ovarian cancer, we compared the AUC between CA125 alone to that of our 5-marker panel, as determined by ROC curves. CA125 alone has an AUC of 0.87. On the other hand, our newly identified 5-marker panel has an AUC of 0.98. Thus, our pilot study has identified a promising set of 5 serum markers for early detection of ovarian cancer.

### Validation of 5-marker panel for detection of ovarian cancer with ELISA assay (Figure 6)

**Figure 6:**
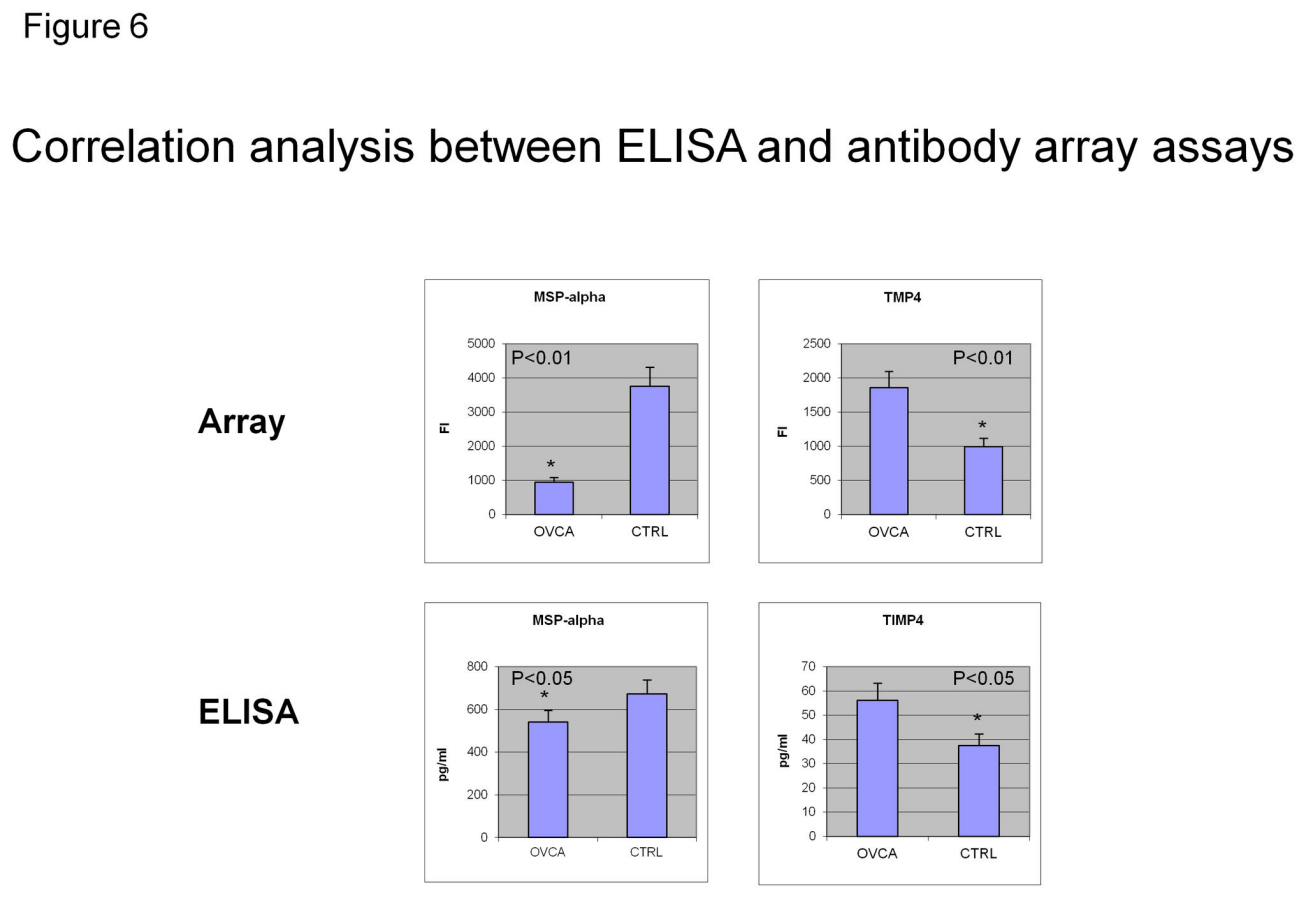
Correlation analysis between ELISA and antibody array assays. Levels of two protein markers (MSP-alpha and TIMP-4) identified as being differentially expressed in ovarian cancer samples using antibody arrays were confirmed with ELISA. The antibody array data were completely concordant with the ELISA data in classifying sera from ovarian cancer patients and healthy controls. Antibody array data are shown as median array signal intensity (FI), and ELISA data are shown as mean protein concentration (ng/ml).

To confirm the multiplex detection of the array data, we performed single-target ELISA assays to quantitatively measure the expression levels of these cytokines individually, and these results were compared with the array data. The relative expression levels for proteins measured by the array and ELISA were similar (see Figure 6). All 4 markers (MSP-alpha, TIMP-4, PDGF-R alpha, and OPG) identified by ANN analysis and split-point score analysis were confirmed by ELISA kits. Figure 6 shows representative data for two of these markers, MSP-α and TIMP-4.

## Discussion

CA125 is one of the most important biomarkers for ovarian cancer. It is often used effectively for monitoring treatment response and detecting recurrence of ovarian cancer. However, CA125 alone is not a useful diagnostic marker for clinical application due to its low specificity; with a reference cutoff value of 35 IU/ml, CA125 showed limited specificity of 50–60% with the sensitivity of >98% for early-stage disease [4-6]. Elevation of CA125 is detectable in about 0.2–5.9% healthy female and 2.2–27.8% patients with benign ovarian diseases [10]. Elevation of CA125 was observed in only 50% of stage I ovarian cancer patients and increased to 90% or above in stage III and IV ovarian cancer patients [11].

Owing to the complexity and heterogeneity of ovarian cancer, it is unlikely that a single biomarker will be able to detect all subtypes and stages of the disease with a high specificity and sensitivity. By searching the literature and other source, Drs. Polanski and Anderson have compile a list of 1261 proteins believed to be differentially expressed in human cancer [12]. Among them, 260 candidate biomarkers are considered as “high-priority” because they have been implicated as potential cancer markers in multiple publications in the literature and because most of them have been reported to be detectable in serum or plasma. We included many of these biomarkers in our antibody-based biomarker screening.

Cytokines are a diverse group of proteins comprised of cytokines, chemokines, growth factors, interferons, adipokines and lymphokines and play many critical roles of physiological and pathological processes. It is also well known that cytokines, chemokines, growth factors, angiogenesis factors, proteases, apoptotic factors, receptors, adhesion molecules and adipokines play important roles in cancer development, progression and metastasis. Growing evidence suggests that a complex cytokine network is involved in ovarian cancer. A number of autocrine and paracrine cytokine loops have been identified in ovarian cancer and influence the biology of this tumor. Detection of expression patterns of multiple cytokines can provide new insights on cancer biology, identify new molecular targets for cancer treatment and discover new biomarkers for diagnosis and prognosis of disease [13,14].

In this study, we have demonstrated the effectiveness of screening a semi-quantitative, sandwich-based antibody array detecting a panel of 174 markers in the serum of 34 ovarian cancer patients and 53 age-matched healthy controls to identify a panel of 5 serum protein markers, including CA125, that can effectively detect ovarian cancer with high specificity (95%) and high sensitivity (100%) with AUC of 0.98. These markers were validated with ELISA assay.

We observed that CA125 alone has an AUC of 0.87, on the other hand, our newly identified 5-marker panel has an AUC of 0.98, indicating improved efficiency when detection of CA125 is combined with other 4 putative protein biomarkers for detection of ovarian cancer (TIMP-4, OPG, PDGF-R alpha, and MSP-alpha).

TIMP-4 belongs to the matrix metalloproteinase (MMP) superfamily. MMPs are essential elements in extraceullular matrix (ECM) degradation, including regulating the release of ECM-bound cytokines and growth factors, which leads to angiogenesis, cellular invasion and, eventually in many cancers, metastasis. These MMPs are tightly controlled and regulated by several TIMPs, several of which appear to play a critical role in tumorigenesis. Chegini’s Lab has reported elevated expression of TIMP-4 in ovarian cancer tissues by IHC analysis, indicating its potential role in tumorigenesis of ovarian cancer [15].

OPG belongs to TNF superfamily and can be linked to the nuclear factor kappa-light-chain enhancer of activated B cells (NFκB) and tumor necrosis factor-related apoptosis inducing ligand (TRAIL) signaling pathways. OPG was first identified by its ability to regulate the homeostasis of bone remodeling. However, Piche’s Lab reported that OPG can serve as such survival factor by protecting TRAIL-induced apoptosis in ovarian cancer cells, indicating its potential role in the development and progression of ovarian cancer [16].

PDGF-R alpha is a receptor in the PDGF superfamily. Serving as angiogenic growth factors, PDGFs play important role in cell growth, chemotaxis, angiogenesis and, in the context of cancer, reconstruction of tumor stromal microenvironments. Jakobsen’s Lab reported that PDGF-R alpha showed higher expression in ovarian cancer tissues in comparison with adjacent normal tissues [17]. It was also reported that PDGF-R alpha is expressed more often in serous carcinomas than in endometriod and mucinous tumors [18], which is consistent with the findings of our study, in which the majority of tumors tested (29 of 34) were serous.

MSP is a growth factor involved in activating macrophage stimulating receptor-1 (MSTR1). The alpha chain of MSP (MSP-alpha) is secreted by cleavage of pro-MSP. There are reports showing that the MSP pathway plays an important role in tumor metastasis [19].

In summary, using 174-marker cytokine antibody array technology, we identified a panel of 5 serum protein markers which can detect ovarian cancer with both high specificity and high sensitivity, indicating its promising application in personalized medicine for ovarian cancer detection. Additionally, considering that a relatively small sample size (N<100) used in this investigation achieved an extremely high sensitivity (100%) and relatively high specificity (95%), we offer some hope that validation of multi-biomarker panel may someday be useful to screen for a deadly cancer that rarely gets diagnosed in its early stages.

Protein and antibody arrays have emerged as a promising technology to study protein expression and protein function in a high throughput manner. These protein and antibody arrays present a new opportunity to profile protein expression levels in cancer patients’ samples and identify useful biosignatures for drug development and patient care. Our 5-marker panel could effectively distinguish ovarian cancers from healthy controls These 5 individual markers are not unique to ovarian cancers, as shown by their expression in other cancer types, including breast cancers [18-22], lung cancers [23], colorectal cancers [24], prostate cancers [25,26], hepatocellular carcinomas [27], pancreatic cancers [28], etc. Therefore, it will be very important and interesting to investigate whether this combination of 5-markers can detect other cancer types as well. Two of most important components in biomarker discovery program are high quality of patients’ samples and high-content screening and high-throughput technologies. Therefore, the combination of proven antibody-based detection technology and platforms from us and well-characterized pre-diagnostic samples from PLCO at National Cancer Institute (NCI) will provide a unique opportunity for biomarker discovery and validation [29,30]. Such investigations will not only serve to validate the specific biomarker panel identified in this study; they will help to validate the use of antibody arrays as a high-throuput approach to identify cancer biomarkers for disease screening and detection.

## Materials and Methods

This protocol has been approved by sterling institutional review board. IRB ID: 3303. The review board is sterling independent service, Inc located in 6300 powers ferry road, suite 600-351, Atlanta, GA 30339. Written consent was obtained when collecting samples from both patients and healthy controls.

### Ethical Statement

Written consent was obtained when collecting samples from both patients and healthy controls.

### Sample collection

The serum samples from 34 patients diagnosed with early-stage (I and II) or late-stage (III and IV) ovarian cancers and 53 age-matched healthy controls included in the study were collected from the affiliated hospital, Sun Yat-sen University. Briefly, about 2 ml of venous blood was drawn from patients. Serum was collected and stored at -80°C until needed. Information about ovarian cancer diagnosis, staging, histology, grade and age was available to us, but the unique patient identifiers, such as name, address, day of birth, was not provided.

### Antibody array technology

Semi-quantitative sandwich-based antibody arrays (RayBio^®^ Human Cytokine Array G-Series 2000) were developed as 3 distinct arrays (Human Cytokine Arrays G6, G7, and G8), each representing a unique set of 54 to 60 antigen-specific antibodies to detect a total of 174 serum markers on a glass slide matrix. A pair of antibodies is required to detect each analyte. Glass slides were printed as 4 or 8 identical sub-arrays consisting of spots of each antigen-specific apture antibody for that array. The corresponding detection antibodies were biotin-labeled and combined as a single cocktail reagent for later use. Printed slides were placed in chamber assemblies to allow for incubation of each sub-array with a different sample. After blocking each sub-array with a blocking buffer, sub arrays were incubated with serum samples. Following extensive washing to remove non-specific binding, the cocktail of biotinylated detection antibodies were added to the arrays. After extensive washing, the array slides were incubated with a streptavidin-conjugated fluor (HiLyte Fluor™ 532, from Anaspec, Fremont, CA). The fluorescent signals were then visualized using laser-based scanner system (GenePix 4200A, Molecular Dynamics, Sunnyvale, CA) using the green channel. To increase the accuracy, two replicates per antibody were spotted, and the averages of the median signal intensities for both spots (minus local background subtraction) were used for all calculations. Through these improvements, we can get a coefficient of variation (CV) of about 10% using our glass slide platform.

### ELISA analysis

ELISA was performed according to the RayBio® ELISA manual (RayBiotech, Inc., Norcross, GA, USA). In brief, pre-coated 96-well ELISA plates with captured antibodies were first blocked using a blocking buffer. Duplicate aliquots (100 microliter per well) of diluted sera and multiple dilutions (i.e., concentrations) of standard protein were loaded onto the ELISA plate. The plates were then incubated for 2 h at room temperature (RT). Unbound materials were washed out, and biotinylated anti-cytokine detection antibody was added to each well. The plates were incubated for 1 h at RT. After washing, 100 microliter of streptavidin-conjugated HRP reagent was added to the wells, and the plate was incubated for 30 minutes at RT. After extensive washing, color development was performed by incubation with HRP substrate. After adding stop solution, the optical density (O.D.) at 450 nm was determined for each well using a microplate reader, and the concentrations of the samples were determined by comparison to the standard concentration curves.

### Data analysis

An adjusted t-test was used to test the significance between protein expression levels in ovarian cancer and healthy control samples. *P* values less than 0.05 were considered to be statistically significant.

To determine the signal threshold, signals from the arrays were measured in the absence of samples (using blocking buffer as a blank) and repeated 10 times. The signals generated using blanks were averaged and the standard deviation (SD) was calculated. Signals with values lower than the average blank signal +2xSD were considered as background.

The data was also analyzed using neural network. This powerful tool allows us the find common protein expression profiles to predict cancer. In phase one study, 80% of samples were randomly assigned to training set and the remaining 20% of samples were used as test set. The advantage of this approach is the success of prediction will become more accurate over time, as more data become available.

The data were also analyzed by split-point score analysis. The split point divides the sample space into two intervals, one for ovarian cancer and one for normal controls. The best split-point score of each marker was chosen to ensure the minimization of misclassified samples. For each marker, a score of 0 was assigned to a sample if it fell in the normal control interval for that marker; a score of 1 was assigned to a sample if it fell in the ovarian cancer interval. Overall, an individual was assigned a score as the sum of these assigned scores for *N* different markers. Therefore, the range of such score was between 0 to *N*. A given threshold (*T*) was chosen to optimally separate ovarian cancer from healthy controls, i.e. a given individual with a total score <*T* is predicted to have normal status, whereas an individual with a total score >*T* was diagnosed as ovarian cancer.

From the above data, we calculated the specificity, sensitivity, positive predictive value (PPV) and negative predictive value (NPV). The ROC was also determined.
